# Laryngotracheal stenosis following intubation and tracheostomy for COVID-19 pneumonia: a case report

**DOI:** 10.1093/jscr/rjaa569

**Published:** 2021-01-18

**Authors:** Rishi Vasanthan, Parviz Sorooshian, Vishnu Sri Shanmuganathan, Muhannad Al-Hashim

**Affiliations:** Department of Head and Neck Surgery, University College London Hospitals NHS Foundation Trust, London, UK; Department of Plastic Surgery, Queen Victoria Hospital NHS Trust, East Grinstead, UK; Faculty of Dentistry, Oral and Craniofacial Sciences, King’s Dental Institute, London, UK; Department of Otolaryngology, East Sussex Healthcare NHS Trust, Eastbourne, UK

## Abstract

Laryngotracheal stenosis (LTS) is a rare but serious condition characterized by narrowing of the airway. Iatrogenic injury from endotracheal intubation or tracheostomy insertion is the most common cause of LTS. We present the first reported experience of managing a patient diagnosed with subglottic stenosis (a subtype of LTS) following previous intubation and tracheostomy for coronavirus disease 2019 (COVID-19). This patient required an urgent surgical tracheostomy and subsequent referral to a tertiary airway surgery unit for definitive treatment, which included microlaryngoscopy, laser excision and balloon dilatation. This case highlights that LTS should be included in the differential diagnosis for patients re-presenting with breathing difficulties after prolonged intubation or tracheostomy for COVID-19. Furthermore, it raises the concern of a rise in the incidence of this condition and an increased burden on the few units specializing in airway surgery.

## INTRODUCTION

Laryngotracheal stenosis (LTS) is a rare yet serious pathology with 4.9 cases per million per year in the UK [1]. Subglottic stenosis, a subtype of LTS, is characterized by fibrosis and narrowing of the subglottic space, which extends from the inferior margin of the vocal cords to the cricoid cartilage. Although numerous causes have been associated with the pathology, iatrogenic injury from endotracheal intubation and tracheostomies remains the most common [2]. As such, previous endotracheal intubation or tracheostomy has been documented in 47 and 36%, respectively, of patients later presenting with LTS [3].

In many patients, surgical intervention is required to improve the patency of the airway. Depending on the level and extent of the stenosis, this may include endoscopic dilatation, stenting, laser surgery, cryosurgery or open surgery involving resection and repair with grafts or end-to-end anastomosis [4].

The coronavirus disease 2019 (COVID-19) pandemic has led to a significant rise in endotracheal intubation and tracheostomy insertion for managing severe respiratory manifestations, with over 1600 tracheostomies performed on COVID-19 patients in the UK during the first wave [5]. As such, there are concerns about the impact of airway interventions for COVID-19 on the incidence of LTS, as raised by the European Laryngotracheal Stenosis Committee of the European Laryngological Society [6].

We present the first reported experience of managing a patient who was diagnosed with subglottic stenosis following previous intubation and percutaneous tracheostomy for COVID-19.

## CASE REPORT

This 71-year-old female presented to the emergency department of a District General Hospital with breathlessness and added airway sounds, both of which had gradually worsened over the last month. There were no systemic or infective symptoms, and her voice and swallowing were normal.

She had recently been discharged from hospital 36 days earlier following a 35-day admission with COVID-19 pneumonia and pneumonitis. During her previous admission, she had been intubated for 14 days, after which a percutaneous tracheostomy was inserted for ventilatory weaning. She was decannulated 7 days later. Past medical history included asthma, hypertension and sickle cell trait.

On examination, the patient was comfortable at rest with no respiratory distress, but a low-pitched inspiratory stridor was present. Auscultation revealed a widespread expiratory wheeze. On minimal exertion, she became dyspnoeic with a more prolonged inspiratory and mild expiratory stridor. Observations showed oxygen saturations of 98% on room air, respiratory rate of 20, heart rate of 70, temperature 36.4°C and a blood pressure of 121/80.

**Figure 1:**
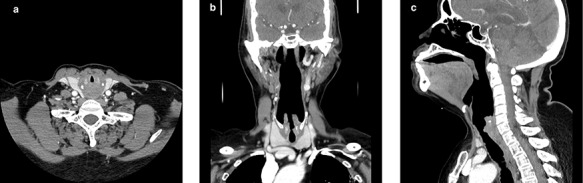
Computerized tomography images of the neck showing stenosis, worst at the subglottic level; (**a**) axial plane; (**b**) coronal plane; (**c**) sagittal plane.

Inflammatory markers were reassuring (white cell count 9.17 and C-reactive protein 17) and an initial chest X-ray showed an improving reticulonodular pattern compared with previously. A swab was negative for COVID-19. A computerized tomography (CT) scan of the neck revealed thickening and oedema from the supraglottic region down to the subglottic region (Fig. 1). The narrowing at the subglottic region was ~4.6 mm on CT compared with a pre-COVID-19 diameter of 10.3 mm on CT. At the narrowest point, there was an approximate cross-sectional obstruction of 80%, which would be consistent with a grade 3 obstruction according to the Cotton–Myer classification [7]. Flexible nasendoscopy (FNE) confirmed a circumferential subglottic stenosis.

Initial management included intravenous dexamethasone and antibiotics, nebulized salbutamol and adrenaline. The patient was moved to the critical care unit for monitoring with a tracheostomy set by the bed. However, further clinical review showed that the patient’s stridor had not improved and oxygen saturations were dropping below 95% on talking or moving. The decision was made to take the patient to theatre for an urgent surgical tracheostomy. This was performed under local anaesthetic due to concerns of difficult endotracheal intubation on induction. This was an uncomplicated procedure, and an unfenestrated, cuffed, size 9 tracheostomy tube was inserted.

Post-operatively, the patient remained clinically well and was stepped down to the ward on day 5. She completed a course of intravenous antibiotics and weaning dose of intravenous dexamethasone. Repeat COVID-19 swabs as well as a COVID-19 sputum test were all negative. The first tracheostomy tube change was done on day 16, to a fenestrated, cuffed, size 6 tube. A repeat CT neck 20 days post-admission showed minimal change in the stenosis with the presence of suspected granular tissue in the left posterior subglottic space, confirmed on FNE.

The patient necessitated referral to a tertiary airway surgery unit, where she was reviewed as an in-patient transfer; she had a microlaryngoscopy, biopsy of granuloma (confirmed as inflammatory) and balloon dilatation of the subglottic stenosis. She was tracheostomy care trained and discharged home. One month later she returned to the tertiary unit, and a microlaryngoscopy, laser excision, steroid injection and balloon dilatation was successfully performed. After this procedure, she received two doses of intravenous dexamethasone and was successfully decannulated. Her voice and breathing were normal and she is being followed up at the tertiary unit.

## DISCUSSION

This case highlights the ever-emerging sequelae of COVID-19 and its treatment, with both morbidity for patients and a further demand on healthcare resources. With the significant increase in tracheostomies and intubations performed during the COVID-19 pandemic, it is reasonable that a proportion of these will present with LTS in the future.

LTS should therefore be considered in patients re-presenting with breathing difficulties after COVID-19 recovery, especially following endotracheal intubation for >10 days or tracheostomy insertion [2, 3, 6]. If suspected, there should be urgent anaesthetic and otolaryngology reviews, and FNE should be performed, particularly if there is stridor or concern about the upper airway. A CT scan of the neck can be performed if the patient is stable.

As demonstrated in this case, most otolaryngology departments are not equipped to provide definitive treatment for LTS and are therefore limited to managing the acute airway concern before referring onwards to a specialist tertiary unit. As tertiary units for airway surgery are limited across the UK, these departments may be inundated with referrals, potentially lengthening the waiting times for these patients. As airway surgery during COVID-19 is a high-risk procedure for both patient and healthcare staff, careful planning and patient selection for surgery will be a key [8]. 
